# Artery of Percheron Syndrome From a Possible Paradoxical Embolus in the Intensive Care Unit: An Easily Missed Diagnosis in Intubated Patients

**DOI:** 10.7759/cureus.109077

**Published:** 2026-05-18

**Authors:** Vinayak Nirmalan

**Affiliations:** 1 Critical Care, Whangarei Hospital, Whangarei, NZL

**Keywords:** artery of percheron infract, bilatera thalamic infarct, loss of consciousness, medical intensive care unit (micu), paradoxical embolus

## Abstract

The following report describes a case of Artery of Percheron syndrome (AOP syndrome), an infarction involving a rare anatomic arterial variant that provides bilateral blood supply to the medial thalamic regions of the brain. This case occurred in a 55-year-old male patient admitted to the intensive care unit (ICU) with reduced consciousness. The patient required intubation and invasive ventilation in the ICU and was initially thought to be suffering from a drug overdose following a normal CT brain report. On extubation, focal neurological findings on bedside clinical examination prompted an MRI scan, which revealed bilateral thalamic and midbrain infarcts characteristic of Artery of Percheron syndrome. Following commencement of dual antiplatelet therapy, a subsequent transthoracic echocardiogram revealed a patent foramen ovale, which was later confirmed on transesophageal echocardiography, raising the possibility of a paradoxical embolus as the underlying cause of the Artery of Percheron occlusion. This case aims to raise awareness of this condition as a potential cause of loss of consciousness, demonstrate the diagnostic difficulties associated with it, and highlight the importance of neurological examination and a high index of suspicion in cases of reduced consciousness despite a normal CT brain.

## Introduction

Artery of Percheron syndrome (AOP syndrome) is a rare but recognized cerebrovascular pathology that accounts for less than 0.1%-0.3% of ischemic strokes [1]. The pathophysiology of AOP syndrome stems from a vascular anatomic variant, the AOP, a single artery arising from the P1 segment of the posterior cerebral artery that supplies the bilateral paramedian thalamic regions and frequently the rostral aspect of the midbrain [2]. Normally, these thalamic regions are supplied by perforating arterial branches arising bilaterally from both posterior cerebral arteries rather than from a single artery [3]. In the event of AOP occlusion, the resulting ischemia causes dysfunction of the thalamic nuclei, often with disruption of connections to the reticular activating system. The resulting presentation features a hallmark triad of reduced consciousness, cognitive disturbance, and vertical gaze palsy [1]. This vertical gaze palsy stems from the disruption of the thalamo-mesencephalic pathways resulting from bilateral paramedian thalamic infarction. It is a key feature that aids in differentiating reduced consciousness caused by AOP syndrome from toxic or metabolic causes [4]. Another major diagnostic tool in differentiating AOP syndrome from toxic or metabolic causes of reduced consciousness is the lack of resolution of symptoms following correction of metabolic abnormalities and clearance of toxins [4]. The most common causes of blockage of the AOP are listed in the literature as small vessel chronic disease as well as thromboembolism [5,6,7]. Initial CT scanning in AOP syndrome frequently appears normal, often leading to false reassurance and diagnostic delays. Diagnostic ambiguity in AOP syndrome can lead to delays in initiating appropriate treatment and thereby risks worse neurological outcomes. The definitive imaging modality, which frequently yields the diagnosis, is MRI scanning, which typically shows bilateral abnormal signals (hyperintensities) in the paramedian thalamic region, often leading to the characteristic *V sign* [7]. Other typical findings include symmetrical thalamic hyperintensities on diffusion-weighted imaging and corresponding hyperintense signals on T2-weighted and fluid-attenuated inversion recovery (FLAIR) sequences, suggesting established infarction with edema [7]. Some cases of AOP syndrome may also show extension of infarction into the rostral midbrain, with abnormal signals in the periaqueductal gray matter [7]. This case demonstrates not only a relatively classical presentation of AOP syndrome but also the added diagnostic difficulties associated with intubated patients in intensive care.

## Case presentation

The patient in question was a male in his fifties with no significant past medical history and no particular cardiovascular risk factors in terms of lifestyle; he was a nonsmoker who was otherwise active and functionally independent. He was found at home by a relative, snoring and unable to be awakened despite vigorous stimulation. He had been completely well and asymptomatic in the preceding days. An ambulance was called, and the crew arrived to find the patient stable from a cardiorespiratory perspective but significantly neurologically obtunded. On crew arrival (roughly 30 minutes after the patient was found), the patient was noted to have oxygen saturations of 97% on room air, a respiratory rate of 14, a heart rate of 87 beats/minute (sinus rhythm), a blood pressure of 177/86 mmHg, and a temperature of 36.7 °C. From a neurological perspective, he had bilaterally constricted pupils measuring 2 mm with sluggish light reflexes and a Glasgow Coma Scale (GCS) score of 7 (Eye, 1; Verbal, 1; Motor, 5). The decision was made to intubate the patient at the scene due to reduced consciousness and snoring respirations. After an uneventful rapid-sequence induction, the patient was intubated, placed on pressure-controlled ventilation, and transferred to the hospital.

**Figure 1:**
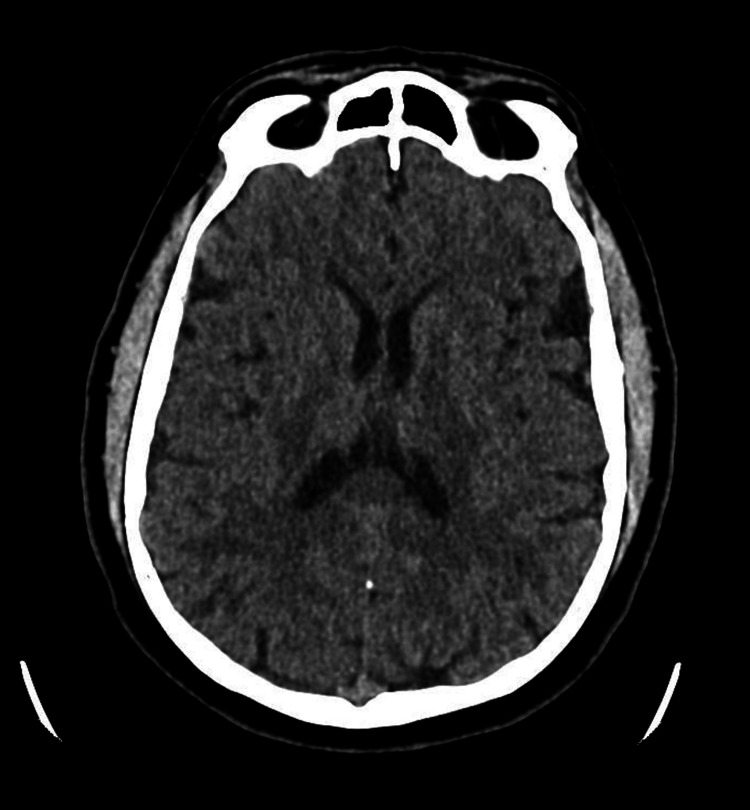
Non-contrast axial CT brain section of the patient on admission, showing no acute pathology. The lateral cerebral ventricles are normal in size, with no features of intracranial hemorrhage, infarction, or trauma.

On arrival to the emergency department, roughly two hours post-crew retrieval, the patient had a full blood panel, which did not show an obvious cause for his presentation. He had a full blood count (Table 1), biochemistry screen including paracetamol and alcohol levels (Table 2), liver function tests (Table 3), and cultures (Table 4). All these results were normal and showed no explanation for his reduced level of consciousness. In addition to this, a urine toxicology screen was performed, looking for amphetamines, benzodiazepines, cannabis, opiates, and cocaine metabolites, all of which came back negative. A CT brain was subsequently performed roughly three hours post-retrieval, which showed no hemorrhages, infarcts, or other acute pathology to explain the presentation (Figure 1). Following this, the patient was admitted to intensive care for ongoing management and diagnostic testing. The timing of arrival to intensive care was three and a half hours after arrival to the emergency department (a total of six hours following discovery of the patient in an obtunded state by his relative).

**Table 1:** Full blood count results and reference ranges.

Full blood count	Normal range	Units	Observed value
Haemoglobin (Male)	130-180	g/L	147
White blood cells (WBC)	4.0-11.0	x10^9/L	7
Neutrophils	2.0-7.5	x10^9/L	4.3
Lymphocytes	1.0-4.0	x10^9/L	1.87
Monocytes	0.2-0.8	x10^9/L	0.66
Eosinophils	0.0-0.4	x10^9/L	0.13
Basophils	0.0-0.1	x10^9/L	0.02
Platelets	150-400	x10^9/L	177
Mean corpuscular volume (MCV)	80-100	fL	91

**Table 2:** Biochemistry panel (including paracetamol and alcohol levels) and reference ranges.

Biochemistry panel	Normal range	Units	Observed value
Sodium (Na⁺)	135-145	mmol/L	138
Potassium (K⁺)	3.5-5.0	mmol/L	4.3
Chloride (Cl⁻)	98-107	mmol/L	106
Bicarbonate (HCO₃⁻)	22-30	mmol/L	24
Urea	2.5-7.8	mmol/L	4.6
Creatinine (Male)	60-110	µmol/L	78
Estimated glomerular filtration rate (eGFR)	≥90	mL/minute/1.73 m²	>90
Calcium (total)	2.10-2.60	mmol/L	2.22
Phosphate	0.8-1.5	mmol/L	0.9
Magnesium	0.7-1.0	mmol/L	0.78
Glucose	3.9-7.8	mmol/L	6
Paracetamol	<22	mg/L	<22
Alcohol	0	mmol/L	0

**Table 3:** Liver function test results and reference ranges.

Liver function test	Normal range	Units	Observed value
Bilirubin (total)	3-20	µmol/L	14
Alanine aminotransferase (ALT)	5-40	U/L	41
Aspartate aminotransferase (AST)	10-40	U/L	25
Alkaline phosphatase (ALP)	30-130	U/L	73
Gamma-glutamyl transferase (GGT)	10-50	U/L	46
Albumin	35-50	g/L	35

**Table 4:** Microbiology test results.

Microbiology test	Result
Blood culture	No growth
Urine culture	No growth

**Figure 2:**
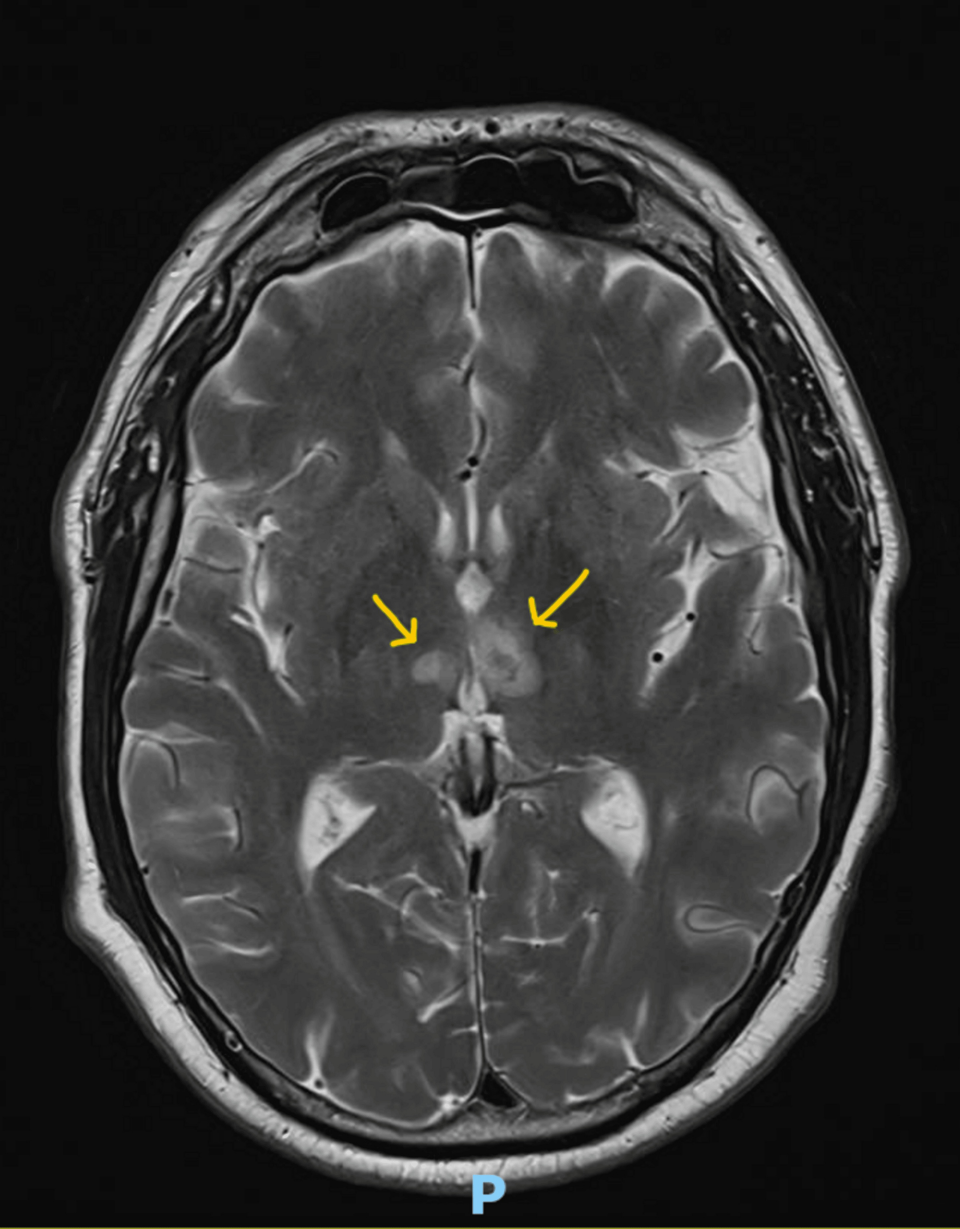
T2-weighted axial MRI imaging of the patient. The gold arrows demonstrate hyperintense signals in the anteromedial thalamic regions bilaterally, suggestive of infarction. These thalamic infarctions form the classical *V sign* associated with AOP syndrome [7]; however, the V shape is less appreciable in this case. AOP, Artery of Percheron

While on intensive care, the patient remained intubated, with normal gas exchange and no cardiovascular support requirements. The question was raised as to whether a lumbar puncture was indicated, given the absence of clear-cut infective symptoms or raised inflammatory markers. The eventual decision was that a lumbar puncture would be useful, based on helping to exclude a subarachnoid hemorrhage as a potential cause of reduced consciousness and also to help rule out an infective cause, as studies have shown that central nervous system infections cannot be definitively ruled out based on a single set of normal serum inflammatory markers [8]. A lumbar puncture was performed roughly 18 hours following admission to intensive care, the results of which were also normal (Table 5). A sedation hold was performed roughly 24 hours into the intensive care admission, during which the patient appeared to be moving all 4 limbs and responding to commands, with adequate tidal volumes. In view of these findings, he was extubated after 1 day. No focal neurological findings were noted during examination when this sedation hold was performed. Following extubation, he was noted on examination to have a vertical gaze palsy, as well as difficulty concentrating and a mild degree of ataxia, which later improved. He was able to hold a conversation but reported difficulty remembering information and thinking clearly, and was not fully oriented to time and place, giving him a Glasgow Coma Scale of 14 (E4V5M6). The rest of his neurological examination showed normal power, tone, sensation, and reflexes in all four limbs. No other cranial nerve defects other than the vertical gaze palsy were noted. In view of these findings, an MRI of the brain was requested. This was performed 48 hours into ICU admission and showed bilateral infarcts in the anteromedial aspects of both thalami. On FLAIR sequences and T2-weighted imaging, bilateral hyperintense lesions were noted in the anteromedial portions of the thalami. Following discussion with a neurologist, the diagnosis of AOP infarction was made, and the patient was commenced on dual antiplatelet therapy with aspirin and clopidogrel. The MRI scans of this patient, showing bilateral thalamic infarcts, are shown in Figure 2.

**Table 5:** Cerebrospinal fluid analysis results and reference ranges. PCR, polymerase chain reaction

Cerebrospinal fluid (CSF) analysis	Normal range	Units	Observed result
Appearance	Clear + colorless	N/A	Clear + colorless
White cells	0-5	Cells/microL	2
Red cells	0	Cells/microL	0
Protein	0.15-0.45	g/L	0.2
Glucose	2.5-4.4	mmol/L	3
Gram stain	No organisms	N/A	No organisms
Viral PCR	No organisms	N/A	No organisms

The patient remained neurologically stable with no further organ support requirements and, therefore, was discharged to a stroke ward on day 3 of his intensive care admission. There, he had a transthoracic echocardiogram (on day 4 of his admission), which revealed a positive bubble test indicative of an intracardiac septal defect - a patent foramen ovale (PFO). This was thought to be a potential route of thromboembolism that led to AOP infarction (a paradoxical embolism in which a venous thrombus passes into the arterial system through an intracardiac right-to-left shunt). His dual antiplatelets were subsequently switched to dabigatran. He was eventually discharged under the care of a rehabilitation team. He still reported issues with cognition, short-term memory loss, and visual disturbances (with ongoing vertical gaze palsy) at the time of discharge, which was day 8 of his admission. An outpatient transesophageal echocardiogram (TOE) was arranged and took place roughly one month following discharge. This scan showed the presence of a PFO, with a bubble test demonstrating definitive right-to-left flow through the PFO. Aside from the PFO, the TOE scan showed an otherwise structurally normal heart.

The TOE findings were used to calculate the patient’s *Risk of Paradoxical Embolism* (ROPE) score - a risk stratification tool used to determine the likelihood of a cerebrovascular event being due to the presence of a PFO [9]. A breakdown of the ROPE score parameters, interpretation, and this patient’s scores are given in Tables 6-7. In the case of this patient, his calculated ROPE score yielded a value of 6, corresponding to an estimated likelihood of the stroke being due to the PFO of 62% [9]. An element of diagnostic uncertainty arose from the fact that this patient had no risk factors for venous thrombosis, in that he had no history of travel, prolonged immobilization, recent infection, dehydration, or any underlying health conditions that generate a prothrombotic state. He had no clinical signs or symptoms of deep venous thrombosis; therefore, further vascular imaging of the lower limbs was not requested in this case. Ultimately, given the TOE findings and ROPE score, it was deemed that paradoxical embolism was a possible, rather than a proven, cause of thalamic infarction.

**Table 6:** Parameters of the Risk of Paradoxical Embolism (ROPE) score and the corresponding score for this patient. The maximum possible score is 10, with higher scores representing a higher percentage likelihood of a paradoxical embolus being the cause of a stroke [9].

ROPE score parameter	Score if the parameter is present	Score for this patient
No history of hypertension	1	1
No history of diabetes	1	1
No history of prior cerebrovascular event	1	1
Nonsmoker	1	1
Superficial cortical infarction on imaging	1	0
Age (years)		
18-29	5	0
30-39	4	0
40-49	3	0
50-59	2	2
60-69	1	0
>70	0	0
Total score	Maximum of 10	6

**Table 7:** Estimated percentage probability of a stroke being related to paradoxical embolism through a PFO for corresponding ROPE scores. Values are based on the original ROPE score development paper published in 2013 [9]. The decrease in paradoxical embolism likelihood seen when moving from a score of 4 to 5 is thought to be a result of sampling variability and noise, as opposed to a clinically meaningful increase in risk. ROPE, Risk of Paradoxical Embolism; PFO, patent foramen ovale

ROPE score	Estimated percentage probability of a stroke being related to paradoxical embolism through a PFO	Overall likelihood of stroke being PFO-related
0-3	0%	Unlikely (PFO likely incidental)
4	38%	Intermediate probability
5	34%	
6	62%	
7	72%	PFO more likely to be stroke-related
8	84%	
9-10	88%	

## Discussion

This case reflects a typical presentation of a rare condition, the presentation and clinical course of which are well reflected in the existing literature and current evidence base [1,2,3]. The incidence of this condition in intensive care in the context of an intubated patient presents additional challenges and raises several key learning points.

Diagnostic difficulty of AOP syndrome

The most prominent difficulty in this case was establishing the initial diagnosis. As with any acute medical emergency, the first priority is stabilizing vital signs and maintaining adequate cardiorespiratory function. This was reflected in this case, as the patient’s level of consciousness and compromised airway meant that intubation and definitive airway management were required before diagnostic testing. The unfortunate consequence of this is that clinical examination is often more difficult in a sedated, ventilated patient, particularly when assessing for focal neurological signs. While the patient displayed fairly typical symptoms of AOP syndrome post-extubation [1,2], the initial period of sedation and ventilation added a diagnostic challenge. The presence of a normal blood panel (while not completely definitive) significantly reduced the likelihood of infection or metabolic disturbances as the underlying cause. Routine toxin screening and blood alcohol levels also made poisoning or overdose an unlikely differential diagnosis. The presence of a normal CT brain and lumbar puncture was useful in ruling out a major acute intracranial hemorrhage and encephalitis; however, these tests did provide an element of false reassurance in terms of excluding intracranial pathology. MRI scanning is frequently listed as the definitive investigation for diagnosing AOP syndrome [7], and this should always be considered in cases of reduced consciousness in the absence of other obvious explanations, particularly in the presence of persistent neurological findings despite normal CT scans and lumbar punctures.

Importance of robust neurological examination

Following extubation, the patient made a significant neurological recovery. He was able to hold a conversation and, while not fully oriented, was able to respond to commands. It would have been relatively easy to attribute the cognitive difficulties to residual effects of sedation and discharge the patient from the ICU based on overall neurological improvement with no organ support requirements. Fortunately, however, a thorough neurological examination was performed, which revealed vertical gaze palsy, ataxia, and short-term memory impairment, all of which are fairly typical features of AOP syndrome [1,2,3] and prompted an MRI scan. With regard to practical neurological assessment of intubated or recently extubated patients, current literature describes a number of frameworks to help mitigate the confounding effects of sedation and paralysis. Scoring systems such as the GCS are less reliable in intubated patients, with current literature highlighting the potentially superior value of the *Full Outline of Unresponsiveness* (FOUR) score [10]. A breakdown of the FOUR score is provided in Table 7. Current literature also supports the use of sequential neurological examinations, focusing on trends rather than individual values, as a gauge of neurological progress [11]. The value of a multimodal approach to neurological examination in critical care patients is also emphasized by the current literature [10,11]. This case highlights the importance of detailed neurological examination even in the presence of a normal CT, particularly in cases of decreased consciousness with a degree of diagnostic uncertainty.

Early consideration of echocardiography and establishing causality between intracardiac shunts and paradoxical embolism

One of the most common causes of AOP syndrome is thought to be thromboembolic events [5,6]. In this case, transthoracic and transesophageal echocardiography showed a PFO, with an overall ROPE score of 6, suggesting a significant possibility that the stroke was due to a paradoxical embolus. This was diagnostically complicated by the fact that no obvious risk factors or clinical signs of venous thrombosis were present. Despite this, paradoxical embolism through the PFO was still thought to be a possible cause of the infarction, as previous cases have shown that clinical and radiological features of deep vein thrombosis may not always be present [12]. In view of this, the patient was listed for surgical repair of the PFO. This case highlights the importance of prompt consideration of transthoracic echocardiography in patients who develop AOP syndrome, with further evaluation using transesophageal echocardiography if defects are detected. Establishing a definitive link between an intracardiac defect and stroke can be difficult, particularly given that many cases of paradoxical embolism are not associated with obvious clinical or radiological features of venous thrombosis [12]. Ultimately, the decision was made to list the patient for PFO repair, largely guided by the ROPE score. This case demonstrates the clinical value of risk stratification tools such as the ROPE score in clarifying the extent of the association between a PFO and stroke.

## Conclusions

This was a rare and initially diagnostically complex case requiring thorough evaluation and investigation. AOP syndrome is seldom seen in intensive care units, and the added challenge of limitations in neurological examination in intubated and sedated patients creates an additional dimension of diagnostic difficulty. The key conclusion from this case is that AOP syndrome should be considered as a differential diagnosis in unexplained loss of consciousness presenting to intensive care. Furthermore, the presence of a normal CT scan should not preclude a detailed neurological examination, and a low threshold for further imaging with MRI should be maintained in these patients. In addition, thromboembolic events should be explored as a potential root cause of AOP syndrome in ICU patients, and echocardiography should therefore be considered early to evaluate for potential cardiac defects. The presence of cardiac septal defects should then prompt risk assessment for paradoxical emboli to determine the need for future surgical closure of the defect. This risk assessment process can be supported by evidence-based risk stratification tools such as the ROPE score.
